# Association between single nucleotide variants and severe chronic pain in older adult patients after lower extremity arthroplasty

**DOI:** 10.1186/s13018-023-03683-y

**Published:** 2023-03-09

**Authors:** Rui Xu, Yinan Jin, Suhong Tang, Wenwen Wang, Yu-E Sun, Yue Liu, Wei Zhang, Bailing Hou, Yulin Huang, Zhengliang Ma

**Affiliations:** 1 Department of Anesthesiology, Affiliated Drum Tower Hospital, Medical School, Nanjing University, No. 321 of Zhongshan Road, Nanjing, 210008 China; 2 Department of Anesthesiology, Nanjing Drum Tower Hospital Clinical College of Nanjing Medical University, Nanjing, 210008 China

**Keywords:** Arthroplasty, Chronic postsurgical pain, Older adult, Osteoarthritis, Single nucleotide variants

## Abstract

**Background:**

Hip or knee osteoarthritis (OA) is one of the main causes of disability worldwide and occurs mostly in the older adults. Total hip or knee arthroplasty is the most effective method to treat OA. However, severe postsurgical pain leading to a poor prognosis. So, investigating the population genetics and genes related to severe chronic pain in older adult patients after lower extremity arthroplasty is helpful to improve the quality of treatment.

**Methods:**

We collected blood samples from elderly patients who underwent lower extremity arthroplasty from September 2020 to February 2021 at the Drum Tower Hospital Affiliated to Nanjing University Medical School. The enrolled patients provided measures of pain intensity using the numerical rating scale on the 90th day after surgery. Patients were divided into the case group (Group A) and the control group (Group B) including 10 patients respectively by the numerical rating scale. DNA was isolated from the blood samples of the two groups for whole-exome sequencing.

**Results:**

In total, 661 variants were identified in the 507 gene regions that were significantly different between both groups (*P* < 0.05), including CASP5, RASGEF1A, CYP4B1, etc. These genes are mainly involved in biological processes, including cell–cell adhesion, ECM–receptor interaction, metabolism, secretion of bioactive substances, ion binding and transport, regulation of DNA methylation, and chromatin assembly.

**Conclusions:**

The current study shows some variants within genes are significantly associated with severe postsurgical chronic pain in older adult patients after lower extremity arthroplasty, indicating a genetic predisposition for chronic postsurgical pain. The study was registered according to ICMJE guidelines. The trial registration number is ChiCTR2000031655 and registration date is April 6th, 2020.

**Supplementary Information:**

The online version contains supplementary material available at 10.1186/s13018-023-03683-y.

## Introduction

Hip or knee osteoarthritis (OA) is one of the main causes of disability worldwide and occurs mostly in the older adults. With an aging global population and the increase in the incidence of obesity, the incidence of OA is expected to further increase [1]. Total hip or knee arthroplasty is the most effective method to treat OA; however, severe postsurgical pain can hinder patients' early mobilization and rehabilitation and may also lead to chronic pain [22, 55]. Chronic postsurgical pain (CPSP) is defined as pain occurring at the incision or surgical area and lasting for > 3 months. The prevalence of chronic pain after all surgeries is approximately 10%, but can be as high as 44% after knee arthroplasty and 27% after hip arthroplasty. Thus, the problem of chronic pain after lower extremity arthroplasty is more significant than that after other operations [12, 37].

Previous studies have shown that the development of chronic pain has complex genetic characteristics [21]. To explore new analgesic molecular targets and develop more effective pain management strategies, many studies have explored the genetic mechanisms of pain in depth from the perspective of single nucleotide polymorphism and family genetics [43]. These variants mainly refer to the variation of a single nucleotide that causes a DNA sequence polymorphism. This is the most common genetic variation in the human genome and may change amino acid sequences, thus affecting the structure and function of proteins, ultimately leading to susceptibility to some diseases [44].

Chronic pain after general hip or knee arthroplasty is a difficult postsurgical complication that affects patients' surgical satisfaction, recovery, and quality of life. When patients with medium to severe pain cannot relieve their pain their physical and mental health and daily life are severely and negatively impacted. The variation in pain between patients suggests that a genetic component is involved. However, to date few studies have been conducted on the relationship between lower arthroplasty and CPSP. Therefore, the current study sequenced whole genome exon variants of older adult patients with severe chronic pain after lower extremity arthroplasty. The susceptibility genes related to CPSP were investigated to provide a direction for the development of new clinical treatment methods and identify potential susceptibility biomarkers for patients with chronic postoperative pain.

## Materials and methods

### Subjects and groups

Elderly patients who underwent lower extremity arthroplasty in Drum Tower Hospital Affiliated to Nanjing University Medical School from September 2020 to March 2021 were selected and followed up for postoperative pain assessment using the numerical rating scale NRS. The elderly osteoarthritis patients selected were all caused by joint degeneration rather than fractures or necrosis caused by other reasons. They were performed operations by the same group of doctors and operation method. All patients signed informed consent and were authorized by the ethics committee of Drum Tower Hospital Affiliated to Nanjing University Medical School (Nanjing, China, No. 2019-270-02). Inclusion criteria: (1) Age ≥ 65 years old; (2) American Society of Anesthesiologists (ASA) classification II–III; (3) Patients undergoing lower extremity arthroplasty; (4) Operation duration ≥ 60 min; (5) Patients recorded in electronic medical record system; (6) Patients agreed to participate in the study and signed the informed consent. Exclusion criteria: (1) With gene deficiency disease; (2) Having history of opioid abuse; (3) Using drugs that induce or inhibit liver isoenzymes (such as carbamazepine, quinidine, ketoconazole, etc.) in 4 weeks before operation; (4) Combined with peripheral neuropathy and psychiatric history, chronic pain and long-term opioid use history; (5) With poor body conditions affecting the perioperative pain evaluation; (6) Patients can’t cooperate and communicate with.

The patient's NRS score being ≥ 4 on the 90th day after operation was identified as having severe CPSP. A total of 10 patients were judged to have severe CPSP (group A). 10 patients hospitalized in the same period without chronic postsurgical pain (NRS score = 0 on the 90th day after operation) were randomly selected as the control group (group B).

### Blood sample collection and DNA extraction

5 ml peripheral blood of the patients was collected from the artery, then the DNA was extracted using the Magnetic Universal Genomic DNA Kit (Tiangen, Beijing, China according to the manufacture’s instruction). The degree of DNA degradation and the presence of RNA and protein contamination were analyzed by agarose gel electrophoresis. We used Qubit 3.0 (Life Technologies) to accurately quantify DNA concentration. DNA samples containing more than 0.6 μg were used to build the database.

### SNV detection

Full exon sequencing (WES 1000 g) was performed by Agilent's liquid chip capture system. Genomic DNA extracted from peripheral blood for each sample was fragmented to an average size of 180–280 bp and subjected to DNA library creation using established Illumina paired-end protocols. The Agilent SureSelect Human All ExonV6 Kit (Agilent Technologies, Santa Clara, CA, USA) was used for exome capture according to the manufacturer’s instructions. The Illumina Novaseq 6000 platform (Illumina Inc., San Diego, CA, USA) was utilized for genomic DNA sequencing in Genechem Bioinformatics Technology Co., Ltd (Beijing, China) to generate 150-bp paired-end reads with a minimum coverage of 10 × for ~ 99% of the genome (mean coverage of 100 ×). After sequencing, base call files conversion and demultiplexing were performed with bcl2fastq software (Illumina). The resulting fastq data were submitted to in-house quality control software for removing low quality reads, and then were aligned to the reference human genome (hs37d5) using the Burrows-Wheeler Aligner (bwa), and duplicate reads were marked using Sambamba tools. ANNOVAR software was used to annotate the variants.

Filtering of rare variants was performed as follows: (1) variants with a MAF less than 0.01 in 1000 genomic data (1000g_all), esp6500siv2_all, gnomAD data (gnomAD_ALL and gnomAD_EAS) and in house Genechem-Zhonghua exome database from Genechem; (2) Only SNVs occurring in exons or splice sites (splicing junction 10 bp) are further analyzed since we are interested in amino acid changes. (3) Then synonymous SNVs which are not relevant to the amino acid alternation predicted by dbscSNV are discarded; The small fragment non-frameshift (< 10 bp) indel in the repeat region defined by RepeatMasker are discarded. (4) Variations are screened according to scores of SIFT, Polyphen, MutationTaster and CADD software. The potentially deleterious variations are reserved if the score of more than half of these four software support harmfulness of variations. Sites (> 2 bp) did not affect alternative splicing were removed.

### Statistical analysis

SPSS 22.0 and R software were used for statistical analysis. Normal distribution of continuous data was assessed, which conformed to normal distribution was expressed as mean ± standard deviation ($\overline{x}$ ± SD), while others were expressed as M (Q). Categorical data was statistically described by frequency. Student’s *t*-test or Mann Whitney *U* test was used to compare continuous data of the two groups (age, weight, BMI), and categorical data of the two groups (gender, hypertension or not, diabetes or not) was compared using chi-squared test or Fisher test. Binary logistic regression analysis was used to evaluate the correlation between each variant loci and the risk of chronic pain in patients after lower extremity arthroplasty. Level of *P* values less than 0.05 (two-sided) was regarded as statistically significant in all tests. The FDR correction method was used to test multiple hypotheses on the data, but the sample size was small, so the corrected *P* value had no reference significance. The cluster profiler package of R language was used for gene ontology (go) enrichment analysis and Kyoto Encyclopedia of genes and genomes (KEGG) pathway enrichment analysis. The significantly different target genes were selected for annotation (*P* < 0.05), to get the terms and pathways of relevant genes enriched in.

## Results

### Comparison of basic clinical characteristics between the two groups

As shown in Table 1, there were no significant differences between the two groups in terms of sex, age, weight, BMI, and physical conditions, indicating that the patients’ basic characteristics may not have had an impact on CPSP.

**Table 1:** Basic clinical characteristics of two groups

Features	Group (n = 10, respectively)		*P* value
	A	B	
Female sex	5	7	0.650
Age (mean ± SE)	72 ± 5	68 ± 4	0.087
Weight (mean ± SE, kg)	75.5 ± 10.8	68.4 ± 6.9	0.099
Body mass index (mean ± SE, kg/m^2^)	29.1 ± 5.1	27.3 ± 2.4	0.314
Hypertension	5	7	0.650
Diabetes	1	3	0.582

Group A: no postoperative chronic pain group. Group B: severe postoperative chronic pain group

### Variant analysis

After filtering all loci for quality control, the correlation between 339,767 variants and severe chronic pain in patients after lower extremity arthroplasty was analyzed using binary logistic regression (specific analysis results in Additional file 1, the corresponding genes of SNP IDs in Additional file 2, the genotypes of each individual in Additional file 3). We identified 3426 variants that were significantly correlated with the incidence of severe chronic pain (*P* < 0.05). Synonymous variants and variants in introns and intergenic regions were removed from further analysis as they typically have little impact on function. Thereafter, 661 variants in 507 genes with potential functional impacts remained [13]. The variants in the 10 most significant exon regions are presented in Table 2.

**Table 2:** SNV characteristics of the 20 most significant exon regions

CHROM	POS	ID	GeneName	*P* value	Xsquared	REF	ALT	Func
1	47282772	rs2297809	*CYP4B1*	0.001041944	13.73333333	C	T	Exonic
1	47280884	rs4646491	*CYP4B1*	0.001041944	13.73333333	C	T	Exonic
5	131607588	rs4877	*PDLIM4*	0.001272634	13.33333333	G	T	Exonic
2	107073501	rs62152530	*RGPD3*	0.001398075	10.20833333	C	T	Exonic
5	56177743	rs832582	*MAP3K1*	0.001525071	12.97142857	G	A	Exonic
14	57099859	rs1041316	*TMEM260*	0.001625041	12.84444444	G	A	Exonic
19	1827565	rs10415018	*REXO1*	0.001625041	12.84444444	A	G	Exonic
2	20824498	rs2305458	*HS1BP3*	0.001625041	12.84444444	C	T	Exonic
19	1819125	rs2396359	*REXO1*	0.003027555	11.6	T	C	Exonic
19	1811603	rs7250872	*ATP8B3*	0.003027555	11.6	C	T	Exonic
19	48198675	rs13346368	*BICRA*	0.003255945	11.45454545	A	G	Exonic
1	11014118	rs45537241	*C1orf127*	0.003255945	11.45454545	C	T	Exonic
1	11015165	rs75130475	*C1orf127*	0.003255945	11.45454545	A	G	Exonic
1	92327045	rs1805110	*TGFBR3*	0.003565811	0.003565811	G	A	Exonic
21	39671476	rs2230033	*KCNJ15*	0.003565811	0.003565811	G	A	Exonic
3	36897812	rs11712950	*TRANK1*	0.00386592	0.00386592	T	C	Exonic
14	21511497	rs1243469	*RNASE7*	0.004086771	0.004086771	C	T	Exonic
14	21511458	rs1243469	*RNASE7*	0.004086771	0.004086771	G	C	Exonic
11	104877927	rs507879	*CASP5*	0.004086771	0.004086771	T	C	Exonic
4	103611845	rs227368	*MANBA*	0.004185243	0.004185243	C	T	Exonic

Binary logistic regression analysis was used to evaluate the correlation between each SNP loci and the risk of chronic pain in patients after lower extremity arthroplasty. *P* < 0.05 was regarded as statistically significant

### Gene enrichment analysis

**Fig. 1:**
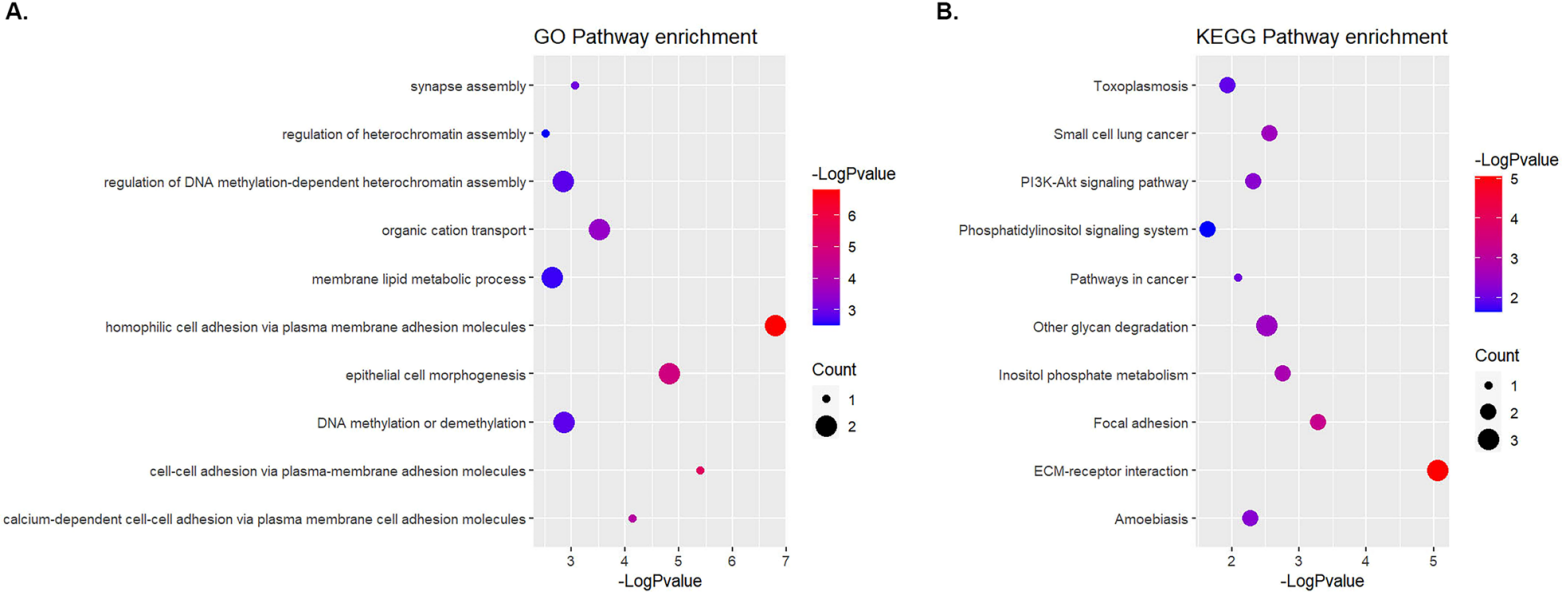
GO and KEGG enrichment analysis. **A** The top 10 categories of GO pathway enrichment analysis. *P* values on the − log10 scale are shown. **B** The top 10 categories of KEGG pathway enrichment analysis. *P* values on the − log10 scale are shown

The genes containing significantly associated variants were subjected to gene ontology (GO) enrichment. Considering a significance threshold cutoff of *P* < 0.05 as the screening standard, 98 GO terms were identified as enriched, including 36 molecular function and 62 biological process terms (Additional file 4). Kyoto Encyclopedia of Genes and Genomes (KEGG) pathway analysis revealed that the significant genes were mainly concentrated in 19 pathways. The top 10 terms and pathways from each analysis were selected for visual representation (Fig. 1). As shown in Fig. 1, susceptibility genes are mostly related to cell–cell adhesion, ECM–receptor interaction, metabolism and secretion of bioactive substances, ion binding and transport, regulation of DNA methylation, chromatin assembly, focal adhesion, and PI3K-Akt.

## Discussion

In the current study, we identified 661 variants in 507 genes associated with CPSP. These results were obtained after bioinformatics analysis and require further functional validation. These genes are associated with numerous functions; however, many are related to neuroinflammation, cognitive function, nervous system development, and key metabolic enzymatic activities.

### Identified genes of interest

Caspase 5 gene (*CASP5*), which encodes for a proinflammatory caspase related to inflammasome formation, was significantly associated with CPSP. Recently, Islam et al. found that blood CASP5 levels were markedly increased in patients with neuropathic pain, suggesting that inflammation is involved in the occurrence and development of neuropathic pain [20], which is consistent with the results of our study. The number of *CASP5* variants in the patients with severe CPSP was significantly higher than that in the control group. According to other studies, the upregulation of *CASP5* can promote the progress of OA and increase the sensory input to the central nervous system, thus aggravating pain [2]. Thus, it can be reasonably inferred that the *CASP5* variants in patients with OA may lead to increased *CASP5* expression levels after arthroplasty, which subsequently increases the risk of severe CPSP. *CASP1* of the caspase family was also significantly related to CPSP in our study. Some studies have shown that *NLRP3-CASP1* mediates the promotion of microglia in neuroinflammation. However, whether it regulates the occurrence or development of pain remains to be further verified [48].

Acid-sensitive ion channel 1 gene (*ASIC1*), which is widely expressed in the nervous system, was also significantly associated with CPSP. ASIC1 is a proton-gated cation channel that is activated by extracellular acidification. It has been reported that MiR-485-5p is involved in the development of OA and that reducing the expression of MiR-485-5p can cause inflammatory pain by upregulating *ASIC1*expression. Therefore, MiR-485-5p/*ASIC1* may be a potential target for the treatment of inflammatory pain in patients with OA [45, 51]. Furthermore, *ASIC1* is also expressed in microglia. In inflammation, *ASIC1* promotes microglia migration and releases inflammatory cytokines, such as NOS and COX-2; this indicates that *ASIC1* is involved in neuroinflammatory response [53]. In our study, the *ASIC1* variant was observed in the control group. It can be speculated that this variant interferes with the correct transformation of *ASIC1* into a protein with normal physiological function, thus reducing the risk of CPSP in the control group. The above hypothesis can be evaluated through further functional verification. These findings suggest that blocking *ASIC1* may be a powerful method for the prevention and treatment of chronic pain after lower extremity arthroplasty.

Another significantly associated gene, A disintegrin and metalloproteinase 12 (*ADAM12*), is expressed in T cells in the brain. As a costimulatory molecule for T-helper 1 cell activation, *ADAM12* mediates tissue inflammation and may play a role in the formation and development of pain. The polymorphism of *ADAM12* has also been significantly associated with knee OA [29, 34]. Many of the genes significantly correlated with CPSP in this study are associated with the pathogenesis of arthritis, including *BCAP29*, *IDO2*, *IGFBP1*, *TLR10*, and *TNC*. How these genes regulate and affect chronic pain after arthroplasty requires further exploration [8, 17, 25, 38, 54].

*ALOXE3* encodes epidermal lipoxygenase-3 (eLOX3) and is expressed in the spinal cord. A study demonstrated that peripheral inflammation significantly increases the level of eLOX3 in the spinal cord and the metabolites of eLOX3. These metabolites promote inflammatory pain via inflammatory cytokines [15].

*PLCE1* encodes phospholipase C ϵ-1, which can stimulate the expression of many inflammatory cytokines, including TNF-α, IL-4, and IFN-γ. It can also activate the NF-κB signaling pathway to promote inflammation and may play an important role in inflammatory pain [27]. The above genes are closely related to inflammation, indicating that the formation of CPSP may be attributed to uncontrolled local inflammation. Therefore, adequate perioperative anti-inflammatory and analgesic therapy may help reduce the risk of CPSP.

CPSP is usually multifactorial and is associated with not only postoperative inflammation but also neuropathic factors, such as peripheral nerve injury, and pain regulation disorder in the central nervous system during operation [41]. AKAP12/SSeCKS is a substrate of protein kinase C, which is upregulated when neurons are injured. It is involved in the regulation of astrocyte activation through the SSeCKS–ERK pathway and aggravating neuropathic pain [28]. *MZF1* encodes for a myeloid zinc-finger transcription factor that regulates the transcription process of related proteins. Many studies have shown that *MZF1* plays an important role in neuropathic pain. *MZF1* can regulate the expression of voltage-gated K^+^ channel and *TRPV1* genes, thus enhancing the excitability of dorsal root ganglion (DRG) neurons, thereby strengthening the transmission of pain signals and promoting the development of neuropathic pain [24, 50, 56]. *ZNF382* encodes for zinc-finger protein 382, which is a transcription factor highly expressed in the nervous system. *ZNF382* is persistently downregulated in injured DRG neurons, losing its binding to the silencer upstream of the Cxcl13 promoter, which promotes the transcription of *Cxcl13*. This eventually contributes to the development and maintenance of neuropathic pain [35]. These genes may promote neuropathic pain by regulating the function of key cells or the expression level of key molecules in the nervous system. The susceptibility genes identified in this paper have been previously associated with pain. The mechanisms through which these genes regulate pain indicate that the underlying mechanism of chronic pain after arthroplasty is complex and multifactorial.

Combined with the above-related factors leading to CPSP, *TET1* from the current study is of particular interest. The TET enzyme encoded by *TET1* is essential for brain function. *TET1* participates in the regulation of DNA demethylation, gene expression, synaptic transmission, and memory formation. Its mutation is related to human cognitive dysfunction. After injury, *TET1* is upregulated so that DNA demethylation at the CpG site of *BDNF* and *mGluR5* promoters is mediated by the *TET1* increase, which increases the transcription of *BDNF* and *mGluR5* and promotes the development of abnormal neuropathic pain. *TET1* also regulates the function of astrocytes through Ca^2+^ signaling, thus affecting neuronal development and cognitive function [14, 18, 19, 52]. Moreover, TET enzyme participates in the development and repair of the nervous system as well as the occurrence and development of neuropathic pain; this may be a powerful therapeutic target for chronic pain.

Although many of our findings are not specifically associated with pain, these genes are involved in the development of the nervous system or the repair process after injury, which is likely to play a role in neuropathic pain. For example, *DCC*, which encodes the DCC receptor, is highly expressed in dopamine cells and interacts with Netrin-1, which plays an important role in regulating synaptic development. Its polymorphism is closely related to the susceptibility to emotional disorders, psychosis, and addiction [47]. Similar to our results, polymorphism of *DCC* showed a significant correlation with chronic pain in another study, further supporting the correlation between nervous system development and pain [21]. Similarly, the nebulin family member *LASP1* plays an important role in formation and maintenance of synaptic in the hippocampus in rats [40]. Genetic variants and the expression level of *LASP1* have also been associated with many neurological diseases, such as schizophrenia, autism, and bipolar disorder [11]. In patients with chronic pain, the expression of these genes is upregulated, indicating a potential association between mood and pain. Additionally, *AP1S1* encodes an adaptor protein complex that is related to synaptophysin and the vesicular acetylcholine transporter, which is very important for spinal cord development; *Slitrk2* mediates excitatory synapse formation and transmission; and *GPR50* encodes the G protein coupled receptor 50 that is associated with synaptic plasticity [16, 26, 39]. *CLIP3* regulates astrocyte proliferation and myelination and participates in regeneration after nerve injury [4, 7]. In patients with CPSP, variants in these susceptibility genes may indicate that the patients are more likely to develop an intraoperative nerve injury. These patients may have difficulty repairing the injured neurons or remodeling synapses after injury because of related gene mutations, resulting in severe chronic pain.

Polymorphisms not only affect patients’ sensitivity to pain, but also the large variation in individual efficacy of analgesic drugs. Some genes may encode for the key metabolic enzyme for these drugs. Variants in these genes alter enzyme activity, preventing drug metabolism or transformation into active forms in the body, leading to great differences in the demand for analgesic drugs in different patients. For example, *AMACR*, encoding α-methylacyl-CoA racemase, catalyzes key steps in ibuprofen metabolism [30], while *AOX1* encodes xanthine dehydrogenase, which metabolizes aza- and oxo-heterocycles representing the scaffold of many drugs like morphine and fentanyl [10] and *CYP1A1* encodes cytochrome oxidase and participates in steroid catabolism [46]. In addition to exogenous drugs, some active molecules in the human body also have analgesic effects, such as endogenous palmitoylethanolamide (PEA), which reduces pain by activating PPAR-α. The *NAAA* encodes for the hydrolase of PEA, and variants in *NAAA* may affect the level of PEA, thus affecting the activation of corresponding receptors, causing the biological transformation from acute pain to chronic pain [9].

### Correlation between enrichment analysis results and pain

As mentioned in the results, the enrichment analysis found many pathways, molecular functions, and biological processes associated with CPSP. The most significant findings are discussed in this study. Cell adhesion molecules mediate the migration of leukocytes to the injured tissues and the release of opioids locally (mainly opioids β-endorphins), which produce an analgesic effect, and block adhesion molecules [36]. Other studies have shown that the serum level of soluble intercellular adhesion molecule-1 (sICAM-1) is significantly correlated with the pain intensity of patients, suggesting that sICAM-1 can be used as a biomarker of pain intensity [33]. The cGMP signaling pathway plays an important role in the processing of pain by sensory neurons and dorsal horn neurons [42]. Phosphatidylinositol bisphosphate (*PIP2*) is located at the key convergence point of multiple receptors, ion channels, and signal pathways that promote chronic pain. Downregulating *PIP2* in neurons can weaken receptor signals, which is a potential new method for the treatment of pain [31]. In OA, the degradation of chondrocytes affects the synthesis and secretion of ECM and the degradation of ECM further damages the chondrocytes. Chondrocytes release various proinflammatory cytokines that stimulate inflammation, such as IL-1, IL-6, IL-17, TNF-α, and PGE2, which not only induce pain but also stimulate chondrocytes to secrete protease, thus hydrolyzing ECM and aggravating OA symptoms [23]. The stimulation and destruction of chondrocytes also occurs in the process of arthroplasty. Surgery intensifies inflammation of the operated joint, resulting in severe acute pain. What’s more, the aseptic loosening after arthroplasty also could be a factor of CPSP, which related to aseptic periprosthetic osteolysis. Maffulli et al. [6] found that individual susceptibility to aseptic loosening has a genetic susceptibility component in this condition, which include contributions by many polymorphisms, and genes encoding for proinflammatory cytokines. Intervention of the ECM–receptor interaction may become a therapeutic target to reduce acute postsurgical pain and reduce the transformation of acute pain to CPSP. *PI3K* and its downstream *Akt* are widely expressed in the spinal cord, especially in the lamina I–IV of the dorsal horn, where the primary afferent nerve fibers mostly terminate. At present, many studies have confirmed that the PI3K/Akt pathway plays a key role in the development and maintenance of chronic pain [3]. Our study suggested that the PI3K/Akt pathway also plays a role in chronic pain after arthroplasty and may become a powerful therapeutic target.

## Conclusions and limitations

Pain after total hip or knee arthroplasty has always been of clinical concern as it greatly decreases the quality of life of the patients and increases the consumption of family and social resources. At present, clinicians mostly use multimode analgesia, combining nerve blocks and a variety of analgesic drugs to reduce pain. However, the prevalence of CPSP remains high [5, 32, 49]. With the continued exploration of mechanisms of pain, the roles of genetic factors in pain have garnered more attention. This study analyzed the whole genome exon variants of older adult patients undergoing lower extremity arthroplasty, mining the variants and clustering the genes associated with severe CPSP. This study has certain limitations, including a small sample size, use of a single statistical method, and no functional verification. In subsequent work, functional validations of these genes will be performed. The validation of *CASP5*, *ASIC1*, and *ADAM12* are of particular interest as these nervous system genes are closely related to chronic pain and immunity in patients who undergo hip and knee arthroplasty. Reduction of the occurrence or degree of postoperative chronic pain by regulating these genes would greatly improve the therapeutic effect and the quality of life of older adult patients after lower extremity arthroplasty. Furthermore, the study may provide a basis and reference for further research on CPSP and gene susceptibility. Additionally, certain biomarkers can be explored and new intervention targets predicted to identify high-risk patients with CPSP.

## Supplementary Information

**Additional file 1**. S1. General table of groupAB SNP.

**Additional file 2**. S2. IDs of SNP.

**Additional file 3**. S3. Genotypes of each individual.

**Additional file 4**. S4. Metascape result.

## Data Availability

Raw data is available containing variant ID numbers and locations, genotypes for each individual, and gene expression data. You can contact us to get data if you need.
